# Effect of implementing an effective farrowing accommodation hygiene routine on clinical cases of disease, medication usage, and growth in suckling and weaned pigs

**DOI:** 10.1093/tas/txae095

**Published:** 2024-06-14

**Authors:** Keely M Halpin, Peadar G Lawlor, Elisa A Arnaud, Júlia Teixé-Roig, John V O’ Doherty, Torres Sweeney, Triona M O’ Brien, Gillian E Gardiner

**Affiliations:** Teagasc, Pig Development Department, Animal and Grassland Research and Innovation Centre, Moorepark, Fermoy, Co., Cork, Ireland; Eco-Innovation Research Centre, Department of Science, South East Technological University, Waterford, Ireland; Teagasc, Pig Development Department, Animal and Grassland Research and Innovation Centre, Moorepark, Fermoy, Co., Cork, Ireland; Teagasc, Pig Development Department, Animal and Grassland Research and Innovation Centre, Moorepark, Fermoy, Co., Cork, Ireland; Eco-Innovation Research Centre, Department of Science, South East Technological University, Waterford, Ireland; Teagasc, Pig Development Department, Animal and Grassland Research and Innovation Centre, Moorepark, Fermoy, Co., Cork, Ireland; Food Technology Department, University of Lleida, Lleida, Spain; School of Agriculture and Food Science, University College Dublin, Belfield, Dublin, Ireland; School of Veterinary Medicine, University College Dublin, Belfield, Dublin, Ireland; Teagasc, Food Safety Department, Food Research Centre, Moorepark, Fermoy, Co., Cork, Ireland; Eco-Innovation Research Centre, Department of Science, South East Technological University, Waterford, Ireland

**Keywords:** Swine, hygiene, cleaning and disinfection, bacteria, antibiotics

## Abstract

The few studies that have evaluated hygiene routines in farrowing accommodation to date have focused on pathogen elimination from pens, with little attention paid to pig growth and no information provided on pig health or medication usage. This study aimed to determine if implementation of an optimized farrowing accommodation hygiene routine could improve pig health and growth and reduce medication usage pre- and post-weaning (PW). Forty seven sows were blocked on parity, previous litter size and body weight and assigned to two treatments: T1) Basic hygiene: cold water washing only with minimal drying time; T2) Optimized hygiene: use of detergent and a chlorocresol-based disinfectant with a 6-d drying time. Total bacterial counts (TBC), *Enterobacteriaceae* counts and adenosine triphosphate (ATP) swabs were obtained from different areas within the farrowing pens. Pig growth and medication usage were monitored from birth to slaughter and carcass data were obtained at slaughter. On entry of sows to the farrowing pens, TBC and *Enterobacteriaceae* counts and ATP concentrations were lower on pen surfaces subjected to the optimized compared to the basic hygiene routine (*P* < 0.05). Pre-weaning diarrhea prevalence was lower in pigs born into optimal compared to basic hygiene pens (0 vs. 22%; *P* < 0.001). The number of clinical cases of disease and injections administered to piglets per litter was 75% and 79% less for the optimized compared to the basic hygiene routine, respectively (*P* < 0.001). This led to reductions of 77% (*P* < 0.001) and 75% (*P* < 0.01), respectively in the volume of antibiotics and anti-inflammatories administered per litter in the optimized hygiene group. Pigs from the optimized hygiene treatment were also heavier at weaning (*P* < 0.01) and their average daily gain (ADG) was higher from day 21 to weaning and days 22 to 49 PW (*P* < 0.05). However, these growth improvements did not carry through to the finisher period. In conclusion, implementation of an optimized hygiene routine reduced the bacterial load in farrowing pens, leading to a reduction in diarrhea and clinical cases of disease and therefore, medication usage, in suckling pigs. Pig growth was also improved during the suckling and early PW periods. Based on the results, an easily implementable farrowing room hygiene protocol with demonstrable benefits for pig health, growth, and welfare can be provided to farmers.

## Teaser

Implementation of an optimized hygiene routine in farrowing rooms reduced diarrhea prevalence and clinical cases of disease in suckling pigs, resulting in a reduction in the volume of anti-inflammatories and antibiotics administered and increased piglet growth during the suckling and early post-weaning periods.

## INTRODUCTION

Antimicrobial resistance (AMR) is one of the largest threats to human health and spans the human, animal, plant, food and environmental sectors (World Health Organization, Food and Agriculture Organization of the United Nations and World Organisation for Animal Health, 2022). There is concern that the high use of antibiotics in livestock production could promote the spread of AMR from animals to humans (Ma et al., 2021). Where species-specific data exists, it indicates that the pig industry has the highest rate of antibiotic usage among livestock production systems (O’Neill et al., 2020; UK-VARSS, 2023). However, there has been an increase in restrictions on antibiotic usage in animals in the European Union (EU) since January 2022 (European Commission, 2019). The routine and prophylactic use of antibiotics in groups of animals and via medicated feed is now prohibited, meaning that antibiotics cannot be used to compensate for poor hygiene and poor management (Collignon and McEwen, 2019; Aslam et al., 2021). As well as this, a ban has been put in place on therapeutic levels of in-feed zinc oxide (ZnO) in the EU since June 2022 due to environmental concerns (European Commission, 2017). This means that ZnO can no longer be used as a medicinal agent and is now only permitted in low doses for nutritional purposes. This is all happening at a time of unprecedented increases in litter sizes, which is having a negative impact on piglet health and growth to weaning (Ward et al., 2020).

For all of these reasons, effective alternatives to antibiotics are of high priority for pig producers. Internal biosecurity aims to prevent the spread of disease within the herd (Alarcón et al., 2021). Internal biosecurity measures include hygiene protocols, e.g., boot dips and cleaning and disinfection of pens and holding areas; vaccination; management of sick/diseased animals by means of a hospital/sick bay; and all-in/all-out animal management systems (Alarcón et al., 2021; Laanen et al., 2013; Postma et al., 2017). Improved biosecurity status has been shown by Postma et al. (2017) to increase growth and decrease mortality in finisher pigs and to reduce antibiotic usage across all production stages; however, the effects of internal biosecurity alone were not evaluated and other management strategies were implemented simultaneously. A study by Laanen et al. (2013) did, however, find that internal biosecurity measures were positively associated with weight gain in finisher pigs. Hygiene is one of the most critical elements of internal biosecurity. For example, cleaning and disinfection of animal housing was the internal biosecurity measure most strongly associated with daily weight gain in the study conducted by Laanen et al. (Laanen et al., 2013). Furthermore, herds with higher antimicrobial usage in growing pigs scored lower for cleaning and disinfection in a study conducted by Raasch et al. (2018). Enhanced cleaning and disinfection of finisher pens has also proven effective in reducing pathogens in the pen environment and subsequently preventing the spread of disease between resident animals (Martelli et al., 2017). A study from our group has also shown, albeit in an abattoir lairage, that the sanitization regime used is critical in terms of reducing pen contamination (Walia et al., 2017).

However, there is a lack of information on the effect of hygiene routines in farrowing accommodation, with, to our knowledge, only two studies conducted to date in farrowing rooms (Merialdi et al., 2013; Law et al., 2021). Furthermore, these farrowing room studies and any weaner or finisher room studies performed to date have focused on pathogen elimination, with little/no attention paid to parameters such as pig growth, health or antibiotic usage (Mannion et al., 2007; Hancox et al., 2013; Luyckx et al., 2016; Misra et al., 2020). The aim of this study was therefore to determine the effect of an optimized vs. a basic hygiene routine in farrowing accommodation on total bacterial and *Enterobacteriaceae* counts in the farrowing pens, and on growth, health and antibiotic and anti-inflammatory usage in suckling piglets. The residual effects of improved farrowing accommodation hygiene on post-weaning (PW) pig growth, health and medication usage to target slaughter weight were also evaluated. The basic hygiene routine was used to obtain hygiene conditions representative of those achieved in farrowing pens on commercial Irish pig farms after implementation of cleaning programs. The optimized hygiene routine was chosen based on one previously shown by our group to have efficacy in terms of reducing/eliminating *Salmonella/Enterobacteriaceae* in the lairage environment of a slaughterhouse (Walia et al., 2017).

## MATERIALS AND METHODS

### Ethical Approval

Ethical approval was granted by the Teagasc Animal Ethics Committee (TAEC2020-282) and Waterford Institute of Technology Research Ethics Committee (WIT2021REC010). The project was authorized by the Health Products Regulatory Authority (project authorization no. AE19132/P130). The experiment was conducted in accordance with legislation for commercial pig production set out in the European Communities (Welfare of Farmed Animals) Regulations 2010 and in Irish legislation (SI no. 311/2010).

### Study Design

This study was performed between March and September 2021 at the Teagasc Pig Development Department, Moorepark, Fermoy, Co. Cork, Ireland. Forty seven sows (Large White × Landrace; PIC, Hermitage Genetics, Kilkenny, Ireland) artificially inseminated using semen (Topigs Norsvin Tempo; Premier Pig Genetics Limited, Stradbally, Co. Laois, Ireland) were used in this study which was carried out over two experimental batches. On day 107 of gestation, sows were blocked within farrowing batch on the basis of parity group (gilts; parity 1 and 2; parity 3 to 5; and parity >5), number born alive at the previous farrowing and body weight (BW). Within block, sows were randomly allocated to one of two experimental treatments: T1) Basic farrowing accommodation hygiene routine (*N* = 22 sows) and T2) Optimized farrowing accommodation hygiene routine (*N* = 25 sows).

### Farrowing Accommodation Hygiene Protocols

The basic and optimized farrowing accommodation hygiene protocols were each implemented in one farrowing room containing 14 farrowing pens in each of 2 batches. Eleven sows/litters from the basic protocol were monitored for growth and health per batch (*N* = 22). Microbiological data were collected from seven basic hygiene pens per batch (*N* = 14 pens). The basic hygiene protocol consisted of removing gross organic matter followed by a thorough washing of the wall and floor surfaces of pens with cold water in a top-to-bottom manner using a Triace 13 hp power washer fitted with a turbo nozzle (Triace, Fermoy, Co. Cork, Ireland). No detergent or disinfectant was used and a minimal drying time of 40 h was allowed, without provision of supplementary heat, before sows were introduced into their pens. Sows were not washed or disinfected as part of this protocol. Twelve sows/litters in batch 1 and 13 sows/litters in batch 2 from the optimized protocol were monitored during the study (*N* = 25 pens). Microbiological data were collected from seven optimized hygiene pens per batch (*N* = 14 pens). The optimized hygiene routine consisted of a presoaking stage, during which floor and wall surfaces were wetted down with water once and then allowed to soak for 18 h. A carboxylic acid-based detergent (Blast Off; Biolink Ltd, Sutton Fields, Hull, UK) was applied the next day to the wall and floor surfaces of each pen in the room at a rate of 1% using a detergent application lance connected to a disinfection cart (Meier-Brakenberg, GmbH & Co. KG, Extertal, Germany) and given a contact time of 20 min. Thorough washing of pens with cold water was then carried out in a top-to-bottom manner using a Triace 13 hp Power Washer fitted with a turbo nozzle. The pens were then allowed to dry for 24 h with supplementary heat provided via a blow heater with a heat output of 29.3 kW at a temperature of ~35 °C (Space warmer; Jack Sealey Ltd, Bury St Edmunds, Suffolk, UK). After 24 h, a chlorocresol-based disinfectant (Interkokask; Interhygiene GmbH, Cuxhaven, Germany) was applied to the wall and floor surfaces of each pen in the room at a rate of 3% using the disinfection lance on the disinfection cart. Pens were left to dry for 6 d after disinfectant application with supplementary heat provided by a blow heater (as outlined above) during the first 24 h. As part of this protocol, on entry to the farrowing pens, sows were gently washed with cold water at a low pressure using the lance of the power washer, following which they were thoroughly disinfected with Virkon S (Lanxess, Cologne, Germany) at a rate of 0.5% using a knapsack sprayer (Pressure Sprayer; Draper Tools, Chandler’s Ford, Hampshire, UK). Each hygiene protocol was applied to one entire farrowing room in each of the two production batches with allocation of treatment to room reversed in the second batch.

The labor required to implement each hygiene routine was monitored by recording the time taken to implement each step of the protocol per farrowing pen. This included the power washing of pens for the basic protocol and the presoaking, application of detergent, application of disinfectant and power washing of pens and washing and disinfecting the sows for the optimized protocol. Water usage was measured using a calibrated water meter (Shanxi Solid Industrial Co. Ltd, Taiyuan, Shanxi, China) which was installed on the power washing line. The volume of water used at each step of each routine was recorded per pen. The additional cost of implementing the optimized hygiene routine was calculated from the cost of the detergent and disinfectant per farrowing pen and the disinfectant used per sow. Additional costs, such as those associated with labor, increased electricity usage, and leaving pens idle for 6 d were not included.

### Sample Collection

A total of 28 farrowing pens (*N* = 14 per treatment) were swabbed the day before implementation of each hygiene routine and after, at entry of the sows to the farrowing pens. This was 6 d after disinfectant application for pens that underwent the optimized protocol and ~40 h after washing for pens that underwent the basic protocol. The 14 pens that underwent the optimized protocol were also swabbed on additional occasions as follows; 20 min after disinfectant application, 24 h after disinfectant application and 72 h after disinfectant application. The udders of 14 sows (*N* = 7 per treatment) were also swabbed before and after entry of sows to the farrowing pens. All swabbing was performed as outlined below with sterile sponges pre-hydrated with 10 mL neutralizing buffer (3M, Bracknell, UK), with a 100-cm^2^ grid used for floor and wall samples. All swabs were taken aseptically, with samples placed into sterile stomacher bags on ice, the grid (when used) sterilized with 70% ethanol between samples and gloves changed between each sample.

#### Swabs for microbiological analysis

A total of five samples were collected from each farrowing pen as follows; floor swab from the piglet lying area (2 × 100 cm^2^ swabs pooled); floor swab from the area behind the sow (3 × 100 cm^2^ swabs pooled), wall swab from the area behind the sow (3 × 100 cm^2^ swabs pooled), sow feeder (entire area of 1,880 cm^2^ swabbed, including the inner and outer surfaces) and piglet drinker (entire area of 314 cm^2^ swabbed, including the inner and outer surfaces; Supplementary Fig. S1). Where swabs were taken from pen locations at multiple sampling time points, care was taken to ensure that repeated swabbing of previously swabbed areas did not occur by swabbing adjacent sites within the same pen location. When collecting samples from the sows’ udder, the entire udder surface was swabbed. The area of the sow’s udder swabbed was estimated as 380 cm^2^, i.e., the width of the sponge (3.8 cm) multiplied by the length of the sow’s udder (estimated at 100 cm).

All swab samples were stored at 4 °C and processed within 24 h. Individual and pooled sponge swabs were suspended in 40 mL maximum recovery diluent (MRD; Oxoid, Basingstoke, UK) and homogenized in a stomacher (Stomacher 400 Circulator; Seward Ltd, Worthing, West Sussex, UK) at 260 rpm for 2 min. An aliquot (6 mL) of the sponge-derived liquid was mixed with 6 mL MRD + 40% glycerol (Honeywell International Inc., Morris Plains, New Jersey, USA) to achieve a final concentration of 20% glycerol. This mixture was transferred to a sterile 15 mL centrifuge tube (Scientific Laboratory Supplies, Dublin, Ireland) and stored at −80 °C until microbiological analysis (within 60 d). Preliminary analysis had shown no difference in bacterial counts from freshly analyzed samples and samples frozen in this way for this duration.

#### Adenosine trisphosphate bioluminescence swabs

A total of 28 pens (*N* = 14 per treatment) were also swabbed using adenosine trisphosphate (ATP) bioluminescence swabs (UltraSnap Surface ATP Test, Hygiena, Watford, UK). Swabbing was performed at the same time points outlined above for microbiological analysis, but only after implementation of the hygiene routines, as heavily soiled pen swabs gave inaccurate readings. Different pens were swabbed for microbiological and ATP analysis, to avoid swabbing the same site/grid in the sampling location multiple times.

A total of five swabs were collected from each farrowing pen from the same locations as for microbiological analysis (see Supplementary Fig. S1). Samples were taken in the same way and all sampling areas were the same, except that for floor and wall sites, only one 100 cm^2^ area was swabbed. The swabs were analyzed immediately after being taken, using a hand held luminometer (EnSURE Touch; Hygiena) according to the manufacturer’s instructions. Swabs were inserted into the luminometer and the relative light unit (RLU) reading recorded. The RLU readings were then converted to RLU/cm^2^ and log-transformed for analysis.

### Microbiological Analysis of Swab Samples From Pens and Sows

The swab sample-glycerol homogenates were thawed at room temperature and then kept on ice. One milliliter of the sample was added to 9 mL of MRD and from that, a 10-fold serial dilution was performed in MRD. Relevant dilutions were plated in duplicate as follows: 1) pour-plated on violet red bile dextrose agar (Merck, Darmstadt, Germany), overlaid and incubated at 37 °C for 24 h for *Enterobacteriaceae;* and 2) spread-plated on Petrifilm aerobic count plates (3M Health Care, Minneapolis, MN, USA) and incubated at 37 °C for 48 h for total bacterial count (TBC). Colony forming units (CFU) were counted and the counts multiplied by 2 to account for the initial 1:2 dilution in MRD + glycerol. Duplicate counts were averaged and presented as log_10_ CFU/cm^2^. The CFU/cm^2^ was calculated as: [(Average CFU/plate * 2) * (volume of original suspension (40 mL)] / (Total surface area * dilution factor). The limit of detection was calculated for each pen location sampled and for the sow’s udder. The limit of detection differs for samples taken before and after implementation of the hygiene protocols, as lower dilutions were plated after protocol implementation due to the fact that lower bacterial counts were expected.

### Animal Management

Pigs were inspected daily and any pig showing signs of ill health was treated appropriately. All veterinary treatments were recorded.

#### Pre-weaning

Sows were inseminated and housed during gestation and lactation as described by Arnaud et al. (2023). Briefly, at day 110 of gestation sows were moved into standard farrowing crates in pens (dimension: 2.5 m × 1.8 m) with cast-iron slats under the sow and plastic slats with a water-heated floor pad for the piglets (BigDutchman; Vechta, Germany). Farrowing room temperature was maintained at ~24 °C. Temperature of heat pads was maintained at 38 to 40 °C for the first 2 d after farrowing and reduced by 2 °C each day to 30 °C ~10 d after farrowing. Artificial lighting was provided daily from 0800 to 1630 hours. Litter size was standardized between 24 and 48 h after parturition, with cross fostering conducted within treatment. The final number of piglets remaining in each litter at 48 h post-partum was affected by the rearing capacity of each sow (i.e., the number of available functional teats), the availability of foster sows to take surplus piglets, and the time of farrowing. Piglets’ teeth were clipped within 24 h of birth. On day 5 post-partum, tails were docked and all piglets were injected with 1 mL of iron (Gleptosil, Ceva Santé Animale, Libourne France). Male pigs remained fully intact and piglets were weaned at day ~27 of lactation.

#### Post-weaning

To study the PW residual effects of hygiene treatment in piglets, a subsample of pigs was selected at weaning to monitor their growth to target slaughter weight. Within each treatment group, pigs were formed into single sex groups of 12 pigs of even weight while ensuring that pigs from individual litters were not over-represented within the pen group. Pens were blocked by sow treatment, sex (entire male or female) and weight. Pen groups (*N* = 11 pens for the basic treatment and *N* = 12 pens for the optimized treatment) were moved to weaner accommodation at weaning. Details of housing in the weaner accommodation are as outlined by Arnaud et al. (2023). Pigs were monitored to slaughter, with feed disappearance and pig weight recorded as outlined below.

At day 49 PW, pen groups were moved to finisher accommodation until pigs reached their target slaughter weight. Details of housing in the finisher accommodation are as outlined by Arnaud et al. (2023). Pigs were weighed once just prior to slaughter on ~day 105 PW and feed intake was recorded weekly. Pigs in each treatment group were slaughtered over a 3-wk period when they reached their target slaughter weight of ~120 kg live weight. The heaviest pigs in each pen group were slaughtered during the first week and the remaining pigs in the pen were slaughtered 7 to 14 d later. Pigs were transported 95 km to the abattoir (Dawn Pork & Bacon, Grannagh, Co. Waterford, Ireland) where they were killed by exsanguination after CO_2_ stunning.

### Diet Preparation and Feeding

The ingredient composition and nutrient content of the sow, weaner and finisher diets were as outlined by Arnaud et al. (2023). Diets were formulated to meet or exceed the requirements of pigs, based on the guidelines provided by the National Research Council (National Research Council, 2012) and manufactured in the Teagasc feed mill (Moorepark, Fermoy, Co. Cork, Ireland) in pelleted (3 mm) form. Details of feeding, including the sequence of diets fed, the number of feeds per day, feed delivery systems, and provision of water are described by Arnaud et al. (2023), except that the finisher diet was provided from day 49 PW instead of day 47 PW.

### Data Recording

#### Sow body weight and back fat thickness

Sow BW and back fat (BF) were recorded on day 110 of gestation, at weaning, and at their subsequent service (~day 6 PW), as outlined by Arnaud et al. (2023).

#### Farrowing performance and pre-weaning piglet growth performance

The number of piglets born (total, live, stillborn, and mummified) was recorded for each litter at birth. The weight and sex of each piglet were recorded at birth when each piglet was tagged for identification purposes. Piglets were individually weighed on days 2, 6, 14, 21, and 27 post-partum using an electronic piglet scale (Defender 3000 XtremeW; O’Donovan Engineering, Coachford, Co. Cork, Ireland) and these data were used to determine the litter weight at each weighing, and piglet pre-weaning average daily gain (ADG). Piglet mortality between birth and weaning was also recorded.

#### Post-weaning pig growth performance

Pen groups were weighed on days 6, 14, 21, and 49 PW and individual weights were recorded at day ~105 PW (slaughter) using an electronic scale (EziWeigh 7i; O’Donovan Engineering). Feed intake was recorded weekly on a pen basis from weaning to slaughter. These data were used to determine the ADG, average daily feed intake (ADFI), and gain to feed (G:F) per pen. Pigs were fasted for 15 to 18 h prior to slaughter before weighing.

#### Carcass data

At the abattoir, carcass cold weight of individual pigs was calculated and muscle depth and BF were measured as outlined by Arnaud et al. (2023). Lean meat content, carcass ADG, carcass G:F, and lean ADG were calculated as outlined by Arnaud et al. (2023).

#### Diarrhea scores

Fecal consistency scores were determined on a pen basis at days 7, 14, 21, and 27 post-partum and at days 6, 14, 21, and 28 PW. A four point scoring system (Casey et al., 2007) was used and the average score from five pigs was determined as the average score for each litter/pen. In brief: 0 = normal (dry pelleted feces), 1 = soft (soft with shape), 2 = mild diarrhea (very soft or viscous liquid), and 3 = severe watery diarrhea (watery or with blood) (Casey et al., 2007). The diarrhea prevalence at each time point was determined by considering a fecal score of 2 or greater as indicative of diarrhea for each litter/pen. The overall diarrhea prevalence was then calculated for the pre-weaning period (days 7 to 27) and the early PW period (weaning to day 28 PW).

#### Clinical cases of disease and antibiotic and anti-inflammatory usage pre-weaning and post-weaning

The number of clinical cases of disease observed and the number of injections administered per pen from birth to slaughter was recorded separately for the pre-weaning and PW periods. A clinical case was defined as any pig requiring treatment with medication on one or more occasions. Antibiotic and anti-inflammatory usage were recorded in sows during lactation and in pigs from birth to slaughter (pre-weaning and PW). Animal ID, pen number, product name, product code, dose administered (mL), frequency of administration, date of administration, and reason for use were recorded when an animal was treated. The antibiotic used was procaine benzylpenicillin (Unicillin injection; Agrihealth, Mullaghadun, Co. Monaghan, Ireland) and the anti-inflammatory used was meloxicam (Loxicom injection; Norbrook, Monaghan, Co. Monaghan, Ireland).

### Statistical Analysis

All data, except for the prevalence of diarrhea, were analyzed using the PROCMIXED procedure in the Statistical Analysis Systems (SAS) software package version 9.4 (SAS Institute Inc., Cary, NC, USA). For analysis of bacterial counts (log_10_ CFU/cm^2^) on pen surfaces before and after implementation of the hygiene protocol, the model included treatment as a fixed effect with pen and batch included as random effects. For analysis of the effect of time on bacterial counts during implementation of the optimized hygiene protocol, bacterial counts on pen surfaces was the fixed effect with batch and pen included as random effects. Day of sampling was the repeated variable in the analysis. For the analysis of reductions in bacterial counts (log_10_ CFU/cm^2^) as a result of implementation of hygiene routines, treatment was included as a fixed effect with initial bacterial count (before starting protocol) included as a covariate in the model. Batch was included as a random effect. The farrowing pen was considered the experimental unit and data were presented as log_10_ CFU/cm^2^.

Sow weight, sow BF depth, pre-weaning litter weight, piglet growth, mortality, number cross-fostered, litter size, medicinal usage (mL/litter), number of injections, and clinical cases of disease per litter were analyzed using treatment as a fixed effect and sow/litter as a random effect. For the analysis of sow weight, sow BF depth, medication usage per sow, pre-weaning litter weight and piglet growth; block was also included as a random effect. When significant in the model the number of piglets born alive per litter and mean piglet birth weight were included as covariates. However, in the case of sow weight and sow BF depth, initial weight, and BF depth, respectively, were included as covariates. Day/period of sampling was included as a repeated variable in the analysis of sow weight, sow BF depth and piglet growth. Sow/litter was considered the experimental unit in all cases except for piglet growth where pig nested within sow was considered the experimental unit.

The prevalence of diarrhea in the farrowing accommodation from days 7 to 27 was analyzed using the PROC Genmod procedure in SAS. A fecal score of 2 or greater for a pen group was considered as indicative of diarrhea. Treatment, day, and the associated two-way interactions were included in the model and the pen group was considered the experimental unit.

For the analysis of PW growth, mortality, the number of clinical cases of disease per pen, the number of injections administered per pen, and medication usage per pen; treatment, sex, and their interactions were included in the model as fixed effects. Block was included as a random effect, while weaning weight was included as a covariate when significant in the model. Day/period of growth was the repeated variable and the PW pen group was considered the experimental unit.

For carcass quality parameters, treatment, sex, and their interaction were included as fixed effects and block as a random effect in the model. Weaning weight was included as a covariate for the analysis of carcass weight, carcass ADG, and carcass G:F, when significant in the model. Carcass weight, when significant in the model, was included as a covariate for the analysis of all other carcass quality parameters. Post-weaning pen group was considered the experimental unit.

For all analyses, the appropriate covariance structure, as indicated by the model fit statistics, was fitted to the data. The denominator degrees of freedom were computed using the Satterthwaite approximation. The slice option was used to test for simple effects at each time point. Results are presented in the text and tables as the least square means together with their pooled standard error. Differences between treatments were considered significant for *P* ≤ 0·05, whereas 0.05 < *P* ≤ 0·10 was considered as a tendency. Differences between least square means were investigated using the *t*-test after Tukey adjustment for multiple comparisons.

## RESULTS

### Labor, Cost, and Water Usage Associated With Implementation of Each Hygiene Routine

The time taken to implement each step of each hygiene routine and the water usage and cost associated with each step of each routine, as well as the total time, water usage and cost are presented in Table 1. The time taken to implement the hygiene routine per pen, was 8.5 min longer for the optimized compared to the basic routine. As well as this, the total additional cost of implementing the optimized hygiene routine was €8.95 per pen. However, this additional cost only covers the disinfectants and detergent required and does not take other costs into account, i.e., costs associated with increased labor and increased electricity usage and with leaving pens idle for 6 d. The total water usage per pen (Table 1) was also 47 L higher for the optimized compared to the basic routine.

**Table 1.** Labor and water usage and cost associated with each step of each farrowing accommodation hygiene routine

	Basic hygiene routine^1^	Optimized hygiene routine^2^
**Total time taken to implement** **routine, min/pen**	**23.5**	**32**
Presoak, min/pen	—	2
Detergent application, min/pen	—	2
Power washing, min/pen	23.5	24
Disinfectant application, min/pen	—	2
Washing of sows, min/sow	—	2
**Total cost, €/pen** ^3^	**No additional cost**	**8.90**
Detergent, €/pen	—	0.50
Disinfectant, €/pen	—	8.35
Disinfectant for sows, €/pen	—	0.05
**Total water usage** **, L** **/pen** ^4^	**142**	**189**
Presoak, L/pen	—	18
Detergent application, L/pen	—	14.1
Power washing, L/pen	142	143
Disinfectant application, L/pen	—	13.9
Washing of sows, L/pen	—	1

^1^Basic hygiene routine = washing of pens with cold water, no use of detergent or disinfectant and minimal drying time (40 h).

^2^Optimized hygiene routine = presoak with cold water for ≤18 h, detergent application (contact time of 20 min), washing of pens with cold water, application of a disinfectant 24 h later and pens allowed to dry for 6 d.

^3^Total cost for implementation of the hygiene routine per pen only covers the cost of detergent and disinfectant for use in the pens and Virkon S for sow disinfection. It does not take other costs into account; for example, those associated with the increased labor, increased electricity usage and with leaving pens idle for 6 d drying.

^4^Total water usage for implementation of the hygiene routine per pen including the volume of water used for dilution of detergent and disinfectant for use in the pens and Virkon S for sow disinfection.

### Microbiological and ATP Data From Farrowing Pen and Udder Swabs

#### Total bacterial counts before and after implementation of the hygiene routines

**Figure 1.**
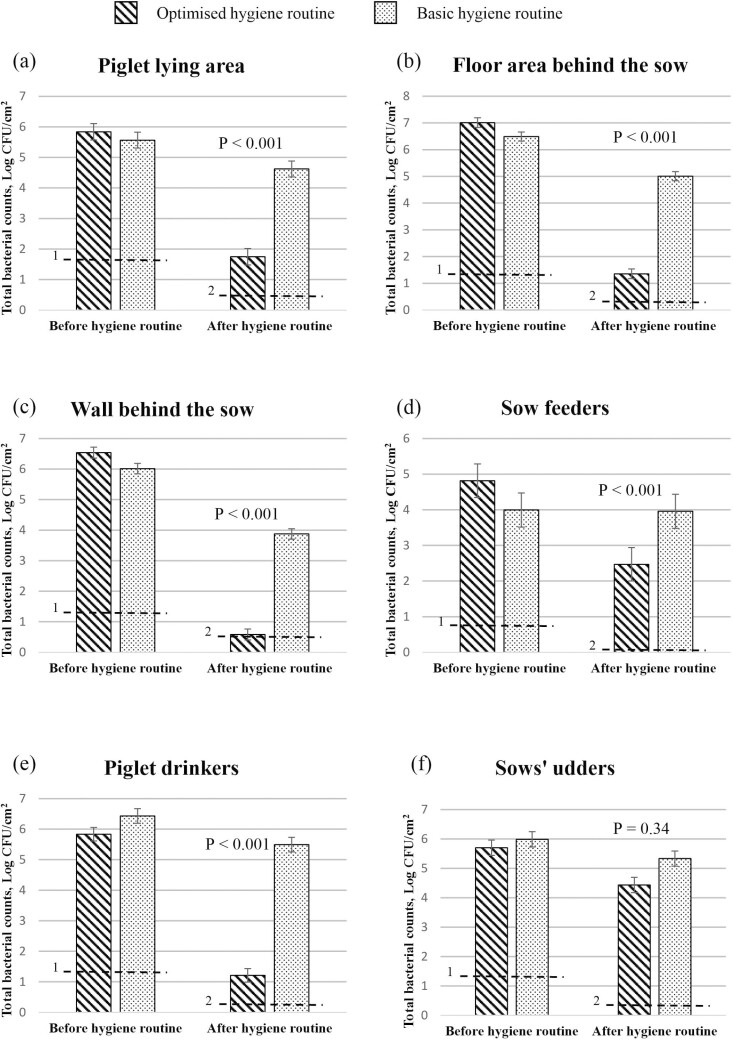
Total bacterial counts from (a) the piglet lying area, (b) the floor area behind the sow, (c) the wall behind the sow, (d) sow feeders, (e) piglet water drinkers, and (f) the sows’ udders. Values are the mean of data from 14 pens and 7 udders per treatment before implementation of the hygiene routine and from 14 pens and 7 udders per treatment after the hygiene routine was implemented (on entry of sows to the farrowing pens). Standard errors of the mean are indicated by error bars. ^1^Limit of detection before implementation of hygiene routine. Values below the limit of detection were recorded as being at the limit of detection. ^2^Limit of detection after implementation of hygiene routine. Values below the limit of detection were recorded as being at the limit of detection. Mean values on entry of sows to the farrowing pens were significantly different between treatments when *P* ≤ 0.05.

The TBC from each sampling location before and after implementation of each hygiene routine are presented in Fig. 1. With the optimized hygiene routine there were 3.11 log_10_ CFU/cm^2^, 5.36 log_10_ CFU/cm^2^, and 5.82 log_10_ CFU/cm^2^ reductions in TBC, respectively, for the piglet lying area, the floor area behind the sow, and the wall behind the sow. Reductions in TBC were less with the basic hygiene routine for all three of these sampling locations (*P* < 0.001), with only 0.48 log_10_ CFU/cm^2^, 1.67 log_10_ CFU/cm^2^, and 2.44 log_10_ CFU/cm^2^ reductions, respectively, observed for the piglet lying area, the floor area behind the sow and the wall behind the sow. As a result, on entry of the sows to the farrowing crates, the TBCs in each of these areas were lower for the optimized compared to the basic hygiene routine (*P* < 0.001). Similarly, on the sow feeders and piglet drinkers there were 2.78 log_10_ CFU/cm^2^ and 4.71 log_10_ CFU/cm^2^ reductions in TBC, respectively, with the optimized hygiene routine. Reductions in TBC were less with the basic hygiene routine for each sampling location (*P* < 0.001), with only ~0.36 log_10_ CFU/cm^2^ and ~0.55 log_10_ CFU/cm^2^ reductions observed, respectively. Subsequently, on entry of sows to the farrowing pens the TBCs on the sow feeders (*P* < 0.01) and the piglet drinkers (*P* < 0.001) were lower for the optimized compared to the basic hygiene routine. However, on entry of the sows to the farrowing pens the TBC on the sows’ udders did not differ between treatments (*P* > 0.05).

#### Enterobacteriaceae counts before and after implementation of the hygiene routines

**Figure 2.**
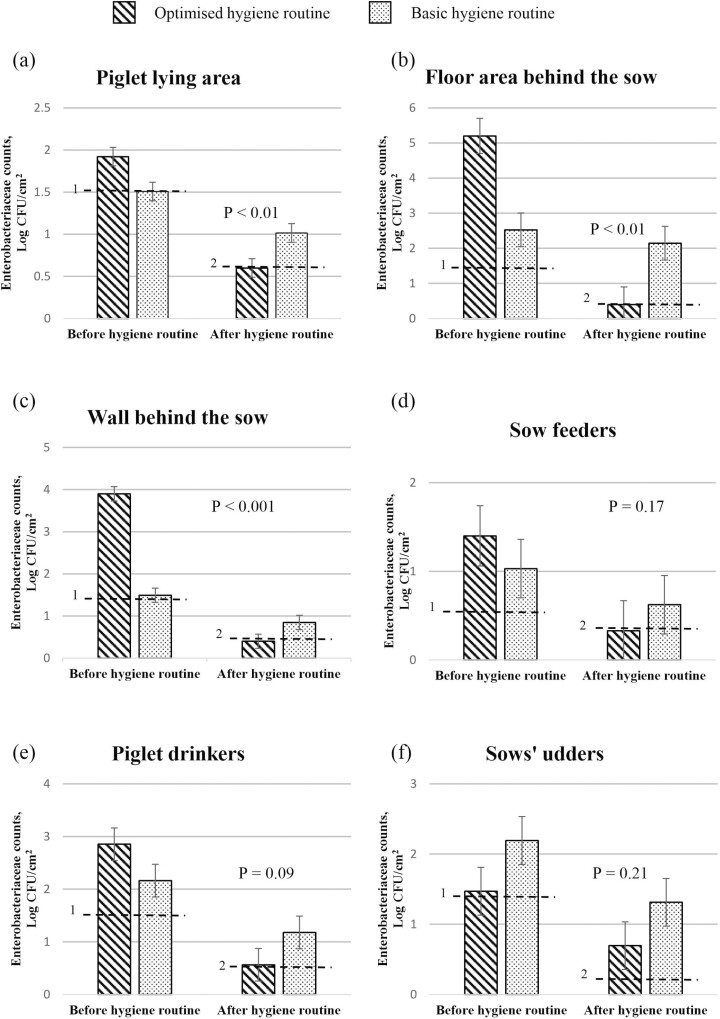
*Enterobacteriaceae* counts from (a) the piglet lying area, (b) the floor area behind the sow (c) the wall behind the sow, (d) sow feeders, (e) piglet water drinkers, and (f) sows’ udders. Values are the mean of data from 14 pens and 7 udders per treatment before implementation of the hygiene routine and from 14 pens and 7 udders per treatment after the hygiene routine was implemented (on entry of sows to the farrowing pens). Standard errors of the mean are indicated by error bars. ^1^Limit of detection before implementation of hygiene routine. Values below the limit of detection were recorded as being at the limit of detection. ^2^Limit of detection after implementation of hygiene routine. Values below the limit of detection were recorded as being at the limit of detection. Mean values on entry of sows to the farrowing pens were significantly different between treatments when *P* ≤ 0.05.

*Enterobacteriaceae* counts from each sampling location before and after implementation of each hygiene routine are presented in Fig. 2. With the optimized hygiene routine there was a 1.15 log_10_ CFU/cm^2^ reduction in *Enterobacteriaceae* counts in the piglet lying area which was higher than the 0.66 log_10_ CFU/cm^2^ reduction obtained with the basic hygiene routine (*P* < 0.05). As a result, on entry of the sows to the farrowing crates, *Enterobacteriaceae* counts in the piglet lying area were lower for the optimized compared to the basic hygiene routine (*P* < 0.05) and were in fact below the limit of detection. With the optimized hygiene routine there were 3.70 log_10_ CFU/cm^2^ and 3.50 log_10_ CFU/cm^2^ reductions on the floor area behind the sow and the wall behind the sow, respectively. Reductions were less with the basic hygiene routine for both sampling locations (*P* < 0.05), with only 1.30 log_10_ CFU/cm^2^ and 0.65 log_10_ CFU/cm^2^ reductions, respectively, observed on the floor area behind the sow and the wall behind the sow. Subsequently, on entry of the sows to the farrowing crate, *Enterobacteriaceae* counts on the floor area behind the sow and the wall behind the sow were lower for the optimized compared to the basic hygiene routine (*P* < 0.001), with both again being below the limit of detection. Reductions in *Enterobacteriaceae* counts tended to be higher for the optimized vs. the basic hygiene routine on the sow feeders (*P* = 0.06). However, on entry of the sows to the farrowing crates, counts on the feeders did not differ between treatments (*P* > 0.05). For the optimized hygiene routine there was a 2.18 log_10_ CFU/cm^2^ reduction in *Enterobacteriaceae* counts on the piglet drinkers which was higher than the 1.33 log_10_ CFU/cm^2^ reduction found with the basic hygiene routine (*P* < 0.01). As a result, on entry of the sows to the farrowing crates, counts on the piglets drinkers tended to be lower (and were below the limit of detection) for the optimized compared to the basic routine (*P* = 0.09). However, *Enterobacteriaceae* counts on the sows’ udders did not differ between treatments (*P* > 0.05).

#### Total bacterial and Enterobacteriaceae counts at interim time points during the optimized hygiene routine

**Figure 3.**
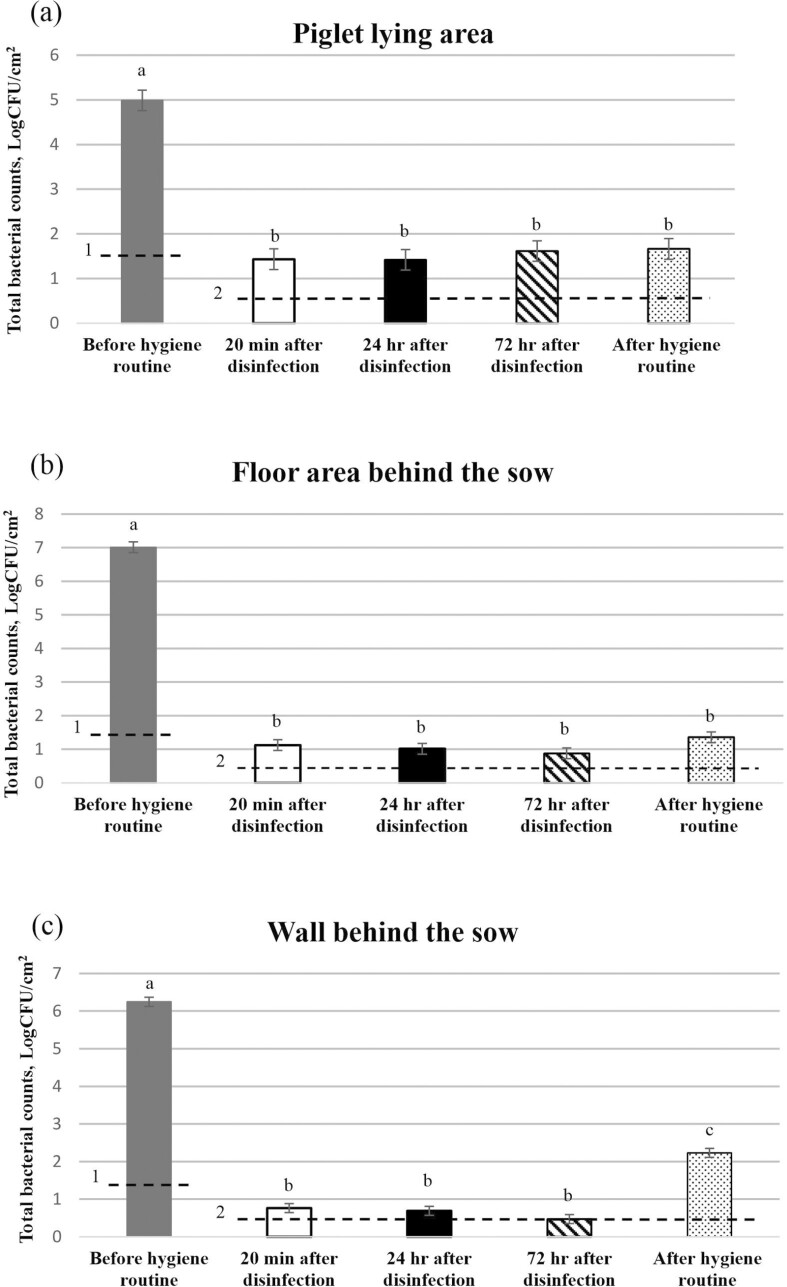
Total bacterial counts from (a) the piglet lying area, (b) the floor area behind the sow, and (c) the wall behind the sow at interim time points during implementation of the optimized hygiene routine and after the entire routine was implemented (on entry of sows to the farrowing pens). Values are the mean of data from 14 pens. Standard errors of the mean are indicated by error bars. ^1^Limit of detection before implementation of hygiene routine. Values below the limit of detection were recorded as being at the limit of detection. ^2^Limit of detection after implementation of each step of hygiene routine and entire routine. Values below the limit of detection were recorded as being at the limit of detection. Bars that do not share a common superscript are significantly different at *P* ≤ 0.05.

**Figure 4.**
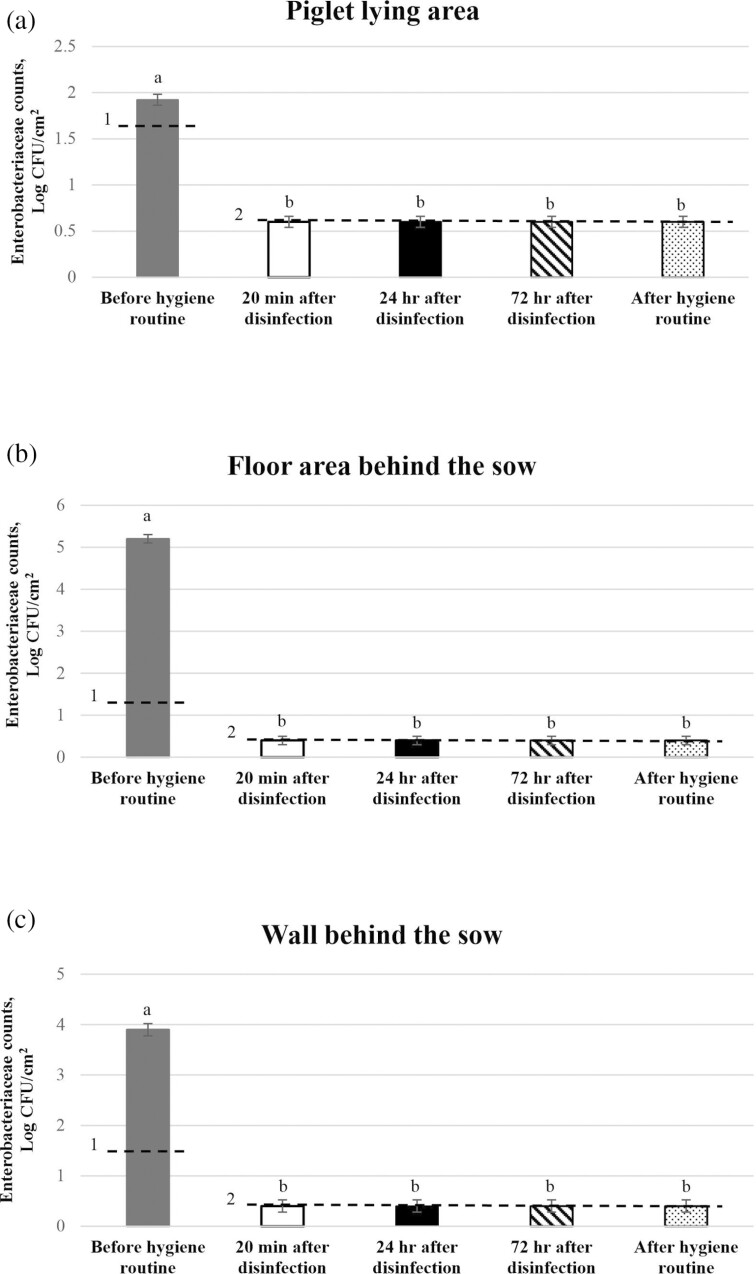
*Enterobacteriaceae* counts from (a) the piglet lying area, (b) the floor area behind the sow, and (c) the wall behind the sow at interim time points during implementation of the optimized hygiene routine and after the entire routine was implemented (on entry of sows to the farrowing pens). Values are the mean of data from 14 pens. Standard errors of the mean are indicated by error bars. ^1^Limit of detection before implementation of hygiene routine. Values below the limit of detection were recorded as being at the limit of detection. ^2^Limit of detection after implementation of each step of hygiene routine and entire routine. Values below the limit of detection were recorded as being at the limit of detection. Bars that do not share a common superscript are significantly different at *P* ≤ 0.05.

Total bacterial and *Enterobacteriaceae* counts at interim time points during implementation of the optimized hygiene routine from three of the sampling locations within the farrowing pens are presented in Figs. 3 and 4, respectively. For both the piglet lying area and the floor area behind the sow, TBC and *Enterobacteriaceae* counts were lower immediately after disinfection (*P* < 0.001), 24 h after disinfection (*P* < 0.001), 72 h after disinfection (*P* < 0.001), and at entry of the sows to the farrowing pens (6 d after disinfection; *P* < 0.001), than prior to implementation of the hygiene routine. However, following disinfection, TBC or *Enterobacteriaceae* counts did not differ between floor samples taken at subsequent sampling time points during the routine (*P* > 0.05). The same was true for the wall area behind the sow, except that the TBC obtained on entry of the sows to the pen was higher than that obtained immediately after disinfection (*P* < 0.001), 24 h after disinfection (*P* < 0.001), and 72 h after disinfection (*P* < 0.001).

#### Adenosine triphosphate readings

The ATP readings from each sampling location after implementation of each of the hygiene routines are presented in Table 2. Adenosine triphosphate concentrations were lower after implementation of the optimized compared to the basic hygiene routine in the piglet lying area (*P* < 0.001), the floor area behind the sow (*P* < 0.001), the wall behind the sow (*P* < 0.001), the sow feeders (*P* < 0.01), and the piglet drinkers (*P* < 0.001).

**Table 2.** Adenosine triphosphate (ATP) concentrations (log_10_ relative light units [RLU]/cm^2^) on farrowing pen surfaces after implementation of each hygiene routine^1^

Area of the pen	Basic^2^	Optimized^3^	SEM	*P* value^4^
Piglet lying area	1.97	0.08	0.068	<0.001
Floor area behind the sow	2.09	0	0.070	<0.001
Wall behind the sow	1.28	0	0.052	<0.001
Sow feeders	1.97	0.73	0.270	<0.01
Piglet drinkers	1.49	0	0.190	<0.001

^1^Least square mean values with their pooled SEM. Values are the mean of data from 14 pens per treatment after the hygiene routine was implemented, i.e., on entry of sows to the farrowing pens.

^2^Basic hygiene routine = washing of pens with cold water, no use of detergent or disinfectant and minimal drying time (40 h).

^3^Optimized hygiene routine = presoak with cold water for ≤18 h, detergent application (contact time of 20 min), washing of pens with cold water, application of a disinfectant 24 h later and pens allowed to dry for 6 d.

^4^Mean values were significantly different between treatments when *P *≤ 0.05.

### Sow Reproductive Performance, Medication Usage, Body Weight and Back Fat Thickness, Cross Fostering, and Pre-Weaning Deaths

Data on sow reproductive performance and piglet mortality are presented in Table 3. The total number of piglets born, total number of live born piglets, total number of stillborn piglets, litter size at 48 h post-partum, and litter size at weaning were not affected by treatment (*P* > 0.05). While 1.3 pigs more were transferred onto litters on the basic compared to the optimized hygiene treatment (*P* < 0.05), there was no effect of treatment on the number of piglets transferred off of each litter (*P* > 0.05) or on the cross-foster balance per litter (*P* > 0.05) at 48 h post-partum. There was no treatment effect on the number of deaths per litter in the first 48 h post-partum (*P* > 0.05) or on the number of deaths from birth to weaning (*P* > 0.05). However, there was a tendency for the number of deaths between 48 h and weaning to increase by 0.5 for the basic compared to the optimized hygiene routine (*P* = 0.08). The mortality rate from birth to weaning was numerically reduced by 28% for the optimized compared to the basic hygiene routine; however, this difference was not significant (*P* > 0.05).

**Table 3.** Effect of hygiene routine on sow reproductive performance, and pre-weaning piglet deaths, diarrhea prevalence, clinical cases of disease and medication usage^1^

	Basic^2^	Optimized^3^	SEM	*P* value^4^
Number of sows	22	25		
Mortality in sows, number^5^	3	2		
Litter				
Total number of piglets born per sow^6^	15.8	16.2	0.73	0.71
Total number of live born piglets per sow	14.9	14.8	0.77	0.98
Total number of stillborn piglets per sow	0.8	1.3	0.49	0.51
Litter size at 48 h post-partum^7^	13.9	13.7	0.30	0.60
Litter size at weaning	12.7	13.1	0.36	0.40
Number of piglets cross fostered off per litter	1.4	0.8	0.27	0.13
Number of piglets cross fostered on per litter	2.3	1.0	0.35	<0.05
Cross-foster balance per litter	0.9	0.2	0.46	0.27
Deaths				
Total number of deaths per litter^8^	2.3	1.6	0.31	0.11
Number of deaths per litter 0 to 48 h^9^	1.3	1.1	0.22	0.56
Number of deaths per litter 48 h to weaning^10^	1.0	0.5	0.22	0.08
Mortality^11^, %	15.1	10.9	2.37	0.22
Diarrhea, clinical cases of disease, injections, and medication				
Diarrhea prevalence, % (days 7 to 27)^12^	22.3	0	0.02	<0.001
Number of clinical cases of disease per litter^13^	1.60	0.40	0.300	<0.01
Number of injections per litter	4.70	1.00	0.900	<0.001
Antibiotic usage per litter, mL	2.34	0.53	0.435	<0.001
Anti-inflammatory usage per litter, mL	0.32	0.08	0.063	<0.01

^1^Least square mean values with their pooled SEM.

^2^Basic hygiene routine = washing of pens with cold water, no use of detergent or disinfectant and minimal drying time (40 h).

^3^Optimized hygiene routine = presoak with cold water for ≤18 h, detergent application (contact time of 20 min), washing of pens with cold water, application of a disinfectant 24 h later and pens allowed to dry for 6 d.

^4^Mean values were significantly different between treatments when *P *≤ 0.05; Mean values tended to be different between treatments when 0.05 ≤ *P* ≤ 0.10.

^5^Mortality in sows: In the Basic group, mortality was due to uterine prolapse (*N* = 2) and an unknown illness (*N* = 1). In the Optimized group, mortality was due to an unknown illness (*N* = 1) and farrowing complications (*N* = 1).

^6^Total number born = number of piglets born alive, stillborn and mummified.

^7^Litter size was standardized within the first 48 h after parturition.

^8^Number of piglets that died between day 0 and day 27 of lactation.

^9^Number of piglets per litter that died within the first 48 h after parturition.

^10^Number of piglets per litter that died between 48 h and day 27 of lactation.

^11^Mortality in piglets: In the Basic group, mortality was due to crushing (*N* = 20), starvation (*N* = 15), unknown reasons (*N* = 11), joint ill (*N* = 5) and splay-leg (*N* = 2). In the Optimized group, mortality was due to crushing (*N* = 13), starvation (*N* = 7), unknown reasons (*N* = 2), splay-leg (*N* = 2), and hemorrhage (*N* = 1).

^12^A fecal score of 2 or greater for a litter was considered indicative of diarrhea at each time point between days 7 and 27. The overall diarrhea prevalence was reported for the pre-weaning period.

^13^Number of pigs per litter requiring treatment with medication during the pre-weaning period on one or more occasions.

Data on sow BW, BF thickness, and medication usage during the suckling period are presented in Supplementary Table S1. There was no effect of treatment on any of these parameters (*P* > 0.05).

### Diarrhea Prevalence, Clinical Cases of Disease, Injections, and Medication Usage in Pigs Between Birth and Weaning

Data on pre-weaning diarrhea prevalence, clinical cases of disease, injections, and medication usage for pigs are presented in Table 3. The pre-weaning prevalence of diarrhea was lower for the optimized compared to the basic hygiene routine (*P* < 0.001). However, it should be noted that a score of 3 (severe diarrhea) was not assigned at any time during the experiment. The number of clinical cases of disease per litter was 75% less for the optimized compared to the basic hygiene routine (*P* < 0.01). The number of injections administered to piglets in each litter was 79% less for the optimized compared to the basic hygiene routine (*P* < 0.001). The volume of antibiotics and anti-inflammatories administered per litter was reduced by 77% (*P* < 0.001) and 75% (*P* < 0.01), respectively, for the optimized compared to the basic hygiene routine.

### Pre-Weaning Pig Growth

Data on piglet weight and growth during the suckling period are presented in Table 4. There was no treatment effect on litter weight (*P* > 0.05) or individual piglet weight (*P* > 0.05) for the overall lactation period or at any time point from birth to day 21. However, at day 27 of lactation (weaning), litter weaning weight was increased by 8.2 kg for the optimized compared to the basic hygiene routine (*P* < 0.01) and individual piglet weaning weight was increased by 400 g (*P* < 0.01). There was a tendency for piglet weight to increase at day 14 for the optimized compared to the basic hygiene routine (*P* = 0.07). There was no overall effect of treatment on piglet ADG from birth to day 27 (weaning) (*P* > 0.05); however, ADG from days 21 to 27 (weaning) increased by 27 g/d for the optimized compared to the basic hygiene routine (*P* < 0.01).

**Table 4.** Effect of hygiene routine on piglet weight and growth during the suckling period^1^

	Basic^2^	Optimized^3^	SEM	*P* value^4^
Number of sows	22	25		
Litter weight, kg				
Day 0 (birth)^5^	21.1	19.8	1.77	0.60
Day 1	20.4	20.2	1.77	0.94
Day 6	31.4	34.0	1.77	0.31
Day 14	54.0	57.4	1.77	0.17
Day 21	78.9	82.7	1.77	0.13
Day 27	103.8	112.0	1.77	<0.01
Overall			1.76	0.27
Mean piglet body weight, kg				
Day 0	1.46	1.45	0.085	0.90
Day 1	1.51	1.51	0.083	0.96
Day 6	2.4	2.5	0.090	0.13
Day 14	4.1	4.3	0.090	0.07
Day 21	6.1	6.2	0.090	0.20
Day 27	8.1	8.5	0.090	<0.01
Overall			0.080	0.20
Average daily gain, g/d^6^				
Days 0 to 1	57	66	8.8	0.44
Days 1 to 6	176	191	7.2	0.12
Days 6 to 14	244	255	7.9	0.27
Days 14 to 21	276	271	7.8	0.67
Days 21 to 27	250	277	7.5	<0.01
Overall			6.8	0.20

^1^Least square mean values with their pooled SEM.

^2^Basic hygiene routine = washing of pens with cold water, no use of detergent or disinfectant and minimal drying time (40 h).

^3^Optimized hygiene routine = presoak with cold water for ≤18 h, detergent application (contact time of 20 min), washing of pens with cold water, application of a disinfectant 24 h later and pens allowed to dry for 6 d.

^4^Mean values were significantly different between treatments when *P *≤ 0.05. Mean values tended to be different between treatments when 0.05 ≤ *P* ≤ 0.10. Total number of piglets born alive per litter was used as a covariate for the analysis of litter weight, piglet weight and piglet average dailly gain between day 1 and day 27 of lactation. Day 27 = average age at weaning.

^5^Total litter weight = weight of piglets born alive and stillborn. Mummified piglets were not weighed.

^6^Piglet average daily gain is calculated on the basis of individual piglet body weight at specific time points during the suckling period/number of days between each time point.

### Post-weaning Pig Growth and Carcass Quality

Data on pig growth and feed intake from weaning to slaughter are presented in Table 5. There was no overall treatment effect on pig weight (*P* > 0.05) and no effect on pig weight at any of the individual time points, except at day 49 PW when there was a tendency for pig weight to be increased by 3.4 kg for the optimized compared to the basic hygiene routine (*P* = 0.06).

**Table 5.** Effect of hygiene routine on post-weaning growth and carcass characteristics^1^

	Basic^2^	Optimized^3^	SEM	*P* value^4^
Number of pens	9	12		
Mortality, number of pigs^5^	0	3		
Off-trial, number of pigs^6^	13	3		
Live weight, kg				
Day 0^7^	9.0	8.6	0.45	0.75
Day 21	15.9	16.1	0.45	0.75
Day 49	32.0	35.4	1.20	0.06
Day 105	127.4	127.4	1.33	1.00
Overall			0.62	0.18
Average daily feed intake, g/d				
Days 0 to 21	472	485	17.7	0.60
Days 22 to 49	1009	1060	36.1	0.34
Days 50 to 105	2575	2641	77.4	0.55
Overall			29.19	0.31
Average daily gain, g/d				
Days 1 to 21	341	359	24.1	0.59
Days 22 to 49	583	687	24.1	<0.01
Days 50 to 105	1138	1099	24.1	0.27
Overall			14.1	0.18
Gain:Feed, kg/kg				
Days 0 to 21	719	726	21.2	0.85
Days 22 to 49	585	641	21.2	0.07
Days 50 to 105	441	418	21.2	0.43
Overall			12.2	0.48
Carcass parameters				
Carcass weight, kg	98.7	97.5	0.91	0.34
Kill-out, %	77.3	76.8	0.58	0.52
Muscle, mm	56.1	55.4	0.86	0.58
Fat, mm	14.5	14.9	0.35	0.42
Lean meat, %	57.9	57.7	0.23	0.60
ADG^8^, g/d	708	697	6.6	0.24
G:F^9^, g/kg	367	356	4.5	0.12

^1^Least square mean values with their pooled SEM.

^2^Basic hygiene routine = washing of pens with cold water, no use of detergent or disinfectant and minimal drying time (40 h).

^3^Optimized hygiene routine = presoak with cold water for ≤18 h, detergent application (contact time of 20 min), washing of pens with cold water, application of a disinfectant 24 h later and pens allowed to dry for 6 d.

^4^Mean values were significantly different between treatments when *P *≤ 0.05; Mean values tended to be different between treatments when 0.05 ≤ *P* ≤ 0.10.

^5^Mortality: Due to sudden death.

^6^Off-trial: One pig from the Basic group was removed due to tail biting on day 49 post-weaning and one pen (12 pigs) from the Basic group was removed on welfare grounds due to tail biting at 66 d post-weaning. Three pigs from the Optimized group were euthanized due to lameness.

^7^D0 = day of weaning.

^8^Carcass average daily gain (ADG; from weaning to slaughter) = [(carcass weight in kg − weaning weight in kg × 0.65) × 1,000]/number of days from weaning to slaughter (Lawlor and Lynch, 2005).

^9^Carcass gain to feed (G:F) (from weaning to slaughter) was calculated as follows: carcass G:F = carcass ADG (g)/daily feed intake (g) (Lawlor and Lynch, 2005).

There was no treatment effect on ADFI for any time period or overall (*P* > 0.05). There was no overall treatment effect on ADG and no effect from weaning to day 21 or day 50 to day 105 PW (*P* > 0.05). However, ADG was increased by 104 g/d for pigs from the optimized compared to the basic hygiene routine from days 22 to 49 PW (*P* < 0.01). From days 22 to 49 PW, the G:F ratio tended to be higher in pigs from the optimized compared to the basic hygiene routine (*P* = 0.07). However, there was no effect of treatment on the G:F ratio at any other time period or overall (*P* > 0.05).

### Diarrhea Prevalence, Clinical Cases Of Disease, Injections, and Medication Usage Between Weaning and Slaughter

The effect of treatment on diarrhea prevalence from weaning to day 28 PW and on PW clinical cases of disease, injections, and medication usage is presented in Supplementary Table S2. Statistical analysis of the effect of treatment on PW diarrhea could not be conducted, as the occurrence of fecal consistency scores indicative of diarrhea (≥2) was rare for both treatments during this period. Nonetheless, compared to pigs from the basic hygiene routine, those from the optimized hygiene routine had a numerically lower prevalence of diarrhea from weaning to day 28 PW (2.4% vs. 7.3%). However, there was no effect of treatment on the number of clinical cases of disease, injections, or medication usage PW (*P* > 0.05).

## DISCUSSION

This study aimed to determine the effects of improving internal biosecurity within farrowing accommodation, by implementing an optimized cleaning and disinfection routine in the farrowing pens. The novelty of the study lies in the fact that as well as investigating the impact of the hygiene routine on the bacterial load of the pen environment, effects on pig health, growth, and medication usage were also determined. As a result of implementing the optimized cleaning and disinfection routine, TBC were reduced in each area of the farrowing pen analyzed compared to when the basic washing routine was used. *Enterobacteriaceae* counts were also reduced (to below the limit of detection) on the floor and wall areas of the pen. These results are in agreement with those from a previous study of ours which used a similar hygiene routine with the same chlorocresol-based disinfectant in the lairage pens of a slaughterhouse (Walia et al., 2017). The efficacy of chlorocresol-based disinfectants is also supported by the findings of in vitro studies (McLaren et al., 2011; Gosling et al., 2017). However, we did not see a significant difference in *Enterobacteriaceae* counts on the piglet drinkers and sow feeders between the basic and optimized hygiene routines. This is in agreement with the fact that feeders and drinkers are highlighted across many studies as the most difficult areas in the pen to clean (Mannion et al., 2007; Heinemann et al., 2020). This is most likely due to their crevices and also the fact that metal surfaces have proven the most difficult on which to obtain bacterial reductions in a previous cleaning study (Hancox et al., 2013). It could also be explained by the occurrence of back splash onto the feeders and drinkers during power washing. Despite this, there was a tendency in the present study for a lower *Enterobacteriaceae* count on the feeders with the optimized regime and this regime also reduced counts on both feeders and drinkers to below the limit of detection. These findings are better than those from other studies, which report that feeders and drinkers often remain contaminated after cleaning and disinfection, or even become more contaminated in some cases (Mannion et al., 2007; Gonzales et al., 2015; Heinemann et al., 2020; Misra et al., 2020). Our results also show that basic cleaning with water alone was not effective in removing total bacteria or *Enterobacteriaceae* from farrowing pen surfaces. This is in agreement with our previous findings from lairage pens (Walia et al., 2017) and is not unexpected as detergent and disinfectant were not applied during the basic protocol.

Adenosine triphosphate is present in all living organisms and so is an indicator of surface hygiene, including both microbial contamination and organic matter (Nantes et al., 2017). Adenosine triphosphate bioluminescence swabs have the advantage of providing real-time data on surface cleanliness without the need for laboratory testing and hence, can be used in field conditions, i.e., on-farm, as in previous studies (Heinemann et al., 2020; Yi et al., 2020). As a result of implementing the optimized hygiene routine in the farrowing accommodation, ATP concentrations were lower in each area of the farrowing pen when compared to those obtained with the basic routine. In this respect, the ATP results follow the same pattern as the bacterial counts. Both Heinemann et al. (2020) and Yi et al. (2020) found that ATP levels correlated well with TBCs from surfaces in finisher and farrowing pens, respectively after cleaning and disinfection. However, Yi et al (2020) showed that this was not true for all sampling sites and both studies found no correlation with counts of fecal indicator bacteria. Despite these limitations, these studies and ours show that ATP swabbing, although requiring investment by farmers, could be a rapid and useful tool to evaluate effectiveness of hygiene routines on-farm. This is particularly relevant, given that a recent study showed that only 1% of pig farms evaluate hygiene after cleaning and disinfection procedures (Makovska et al., 2024).

With the optimized protocol there was no benefit in allowing a drying period of 6 d. This is evidenced by the fact that there were no differences in total bacterial or *Enterobacteriaceae* counts between samples taken 1, 3, or 6 d after disinfection/drying in each area of the pen, except for the TBC on the wall area behind the sow. In fact, in this area counts increased, after 3 d of drying. A possible explanation for this is that after disinfection some bacteria remain in a viable but non-culturable state and that they recover and become culturable (and therefore detectable) during the drying period (Luyckx et al., 2016). Another explanation could be re-contamination with environmental organisms, from dust, for example. Therefore, in the current study, there does not appear to be any benefit in allowing the pens to dry, but if a drying period is allowed, a maximum of 3 d appears optimal. This shorter drying period is also likely to be preferable on most farms, since farrowing pens would be unavailable for use for fewer days. However, it should be borne in mind that only the bacterial load of the pens was assessed and not prevalence of specific pathogens. In a previous study of ours conducted in the lairage of a slaughterhouse, a drying period of 24 to 48 h was important for reducing *Salmonella* prevalence but not *Enterobacteriaceae* counts. Taking all of this into account, we would therefore recommend drying farrowing pens for a period of at least 24 to 48 h but for not more than 3 d. In agreement with our findings, Luyckx et al. (2016) found no benefit of an extended drying period for weaner pens, as TBC was found to be lowest after 4 d of drying, with a numerical increase in counts seen after 7 d which became significant after 10 d. Similarly, Hancox et al. (2013) reported reductions in TBC and *Enterobacteriaceae* counts after 1 d of drying but no additional reductions after 2 or 5 d of drying. It is also noteworthy that in both of these studies, the disinfection processes were not as effective as ours. Luyckx et al. (2016) reported that after disinfection, TBC reduced by just 1.2 Log CFU/625cm^2^ (equates to 0.002 Log CFU/cm^2^), while Hancox et al. (2013) found reductions of only 1.1 to 1.6 Log CFU/cm^2^. This compares with the TBC reductions of 2.78 Log CFU/cm^2^ – 5.82 Log CFU/cm^2^ found in the present study. This difference is likely due to the different hygiene protocols used. Luyckx et al. (2016) used a glutaraldehyde-quaternary ammonium compound-based disinfectant, but no detergent. Similarly, the disinfectant used by Hancox et al. (2013) (Virkon S) also differed from ours, although they used the same detergent. As previously mentioned, chlorocresol-based disinfectants have proven extremely effective in terms of reducing the bacterial load in pens, and therefore, we hypothesize that the greater reduction in bacterial counts achieved in our study is mainly due to the use of this disinfectant.

However, even when only the cost of the disinfectants and detergent were considered, there is an increased cost (€8.90/farrowing pen) associated with the optimized hygiene routine used in the current study compared to the basic hygiene routine. As well as this, water usage and the labor requirement are both higher for the optimized hygiene routine. Although these are important practical considerations, very few cleaning and disinfection studies measure these parameters. However, considering the improved animal health and growth as a result of the effective reduction/removal of bacteria from the pen surfaces found in the current study, the benefits may very well outweigh the cost. However, a full cost-benefit analysis is required in order to determine this.

The current study is one of the first to show that there was no benefit in terms of reduced bacterial counts associated with washing and disinfecting the sows before they entered the farrowing crates. This is likely due to the fact that total removal of soiling from the sows was difficult, as water at a low pressure was used for washing them. It could also be due to the fact that Virkon S was either not effective or not used at a high enough concentration. Virkon S previously performed poorly against *Salmonella* in fecal contamination models (Mclaren *et al*., 2011) and gave varying results in finisher pens, depending on the surface type (Hancox et al., 2013) and this was when used at a rate of 1%, in both cases, compared to the 0.5% used here on the sows. However, we were limited in the choice of disinfectant for use on live animals and the dilution rate permissible. In agreement with our findings, Verhegghe *et al*. (2013) reported no benefit of washing sows using different detergents with different washing protocols in terms of removing methicillin-resistant *Staphylococcus aureus* from the sows’ skin. However, no disinfectant was used. Laanen et al. (2013) found a positive correlation between washing of sows and a reduction of treatment incidences and disease control in suckling piglets. However, no bacterial counts were performed.

As a result of implementing the optimized farrowing room hygiene routine, there was a reduction in the prevalence of pre-weaning diarrhea. There was also a reduction in the number of clinical cases of disease observed per litter, which led to a reduction in the total number of injections and the volume of antibiotics and anti-inflammatories administered per litter from birth to weaning. This is an extremely important finding, as strategies to reduce antimicrobial use are more important now than ever, due to the increased restrictions on antibiotic usage in the EU (European Commission, 2019) and the withdrawal of therapeutic levels of ZnO. The welfare benefits are also important, given the current high priority given to welfare concerns in the EU and elsewhere. The higher use of antibiotics in the basic hygiene group is most likely due to the high microbial load which remained in the pens, causing a number of piglets in this group to develop joint ill, which was the most common clinical case observed during the suckling period. Thereafter, during the early PW period, there were no differences in the number of clinical cases of disease or medication usage between treatments. It should be noted, however, that one pen group of pigs from the basic hygiene treatment was removed from trial due to tail biting and the data on the high usage of antibiotics and anti-inflammatories in this group were not included in the analysis. In agreement with our findings, Raasch et al. (2020) reported a reduction in antimicrobial usage in suckling piglets, but not in weaners or finishers, when a range of tailor-made interventions was implemented at farm level, one of which was improved internal biosecurity. However, the benefit could not be attributed to the improved biosecurity measures alone. Similarly, Laanen et al. (2013) reported that internal biosecurity scores were negatively associated with antimicrobial treatment incidence, suggesting that improved biosecurity might help in reducing antimicrobial use. However, cleaning and disinfection was not one of the internal biosecurity parameters identified as impacting antimicrobial usage. Another study linked reduced antimicrobial usage with cleaning and disinfection, in that herds with higher antimicrobial usage, albeit in growing pigs, scored lower for cleaning and disinfection (Raasch et al., 2018). Overall, ours is the first study to date to show a direct relationship between improved cleaning and disinfection of farrowing rooms and antimicrobial usage and health in suckling pigs.

There was a pre-weaning growth benefit as a result of implementing the optimized hygiene routine, with pigs heavier at weaning compared to pigs born into pens that underwent the basic hygiene routine. Pigs in the optimized hygiene group also had a tendency to be heavier at the end of the weaner period (day 49 PW). It is highly likely that pigs from pens that underwent the optimized hygiene routine were healthier than those in pens from the basic routine since there was a lower number of clinical cases of disease and reduced medication usage recorded in pigs from the former routine. Therefore, these pigs did not have to allocate as much energy towards raising an immune response against the bacterial load in the environment, leaving more available for growth. This is backed up by the fact that Le Floch et al. (2006) showed that pigs weaned into un-sanitized pens had poorer growth and nutrient utilization and higher levels of immune activation PW than those housed in pens that had been cleaned and disinfected. To our knowledge, only one other study to date has investigated the impact of improved farrowing room hygiene on pre-weaning and PW pig growth. In agreement with our findings, they also found that weaning weights and PW ADG were increased for piglets born into farrowing pens which were washed and disinfected compared to those housed in pens washed only with hot water (Law et al., 2021). They also showed that piglets born into disinfected pens had lower fecal and nasal bacterial diversity at birth and that differentially abundant taxa present after birth persisted until weaning. Therefore, analysis of the microbiota of piglets born into sanitized environments is warranted in future studies.

The pre-weaning and early PW improvements in growth observed in pigs from the optimized hygiene group did not persist into the finishing period. Removal of one pen group of pigs on ethical grounds from the basic hygiene group at day 66 PW due to tail-biting likely contributed to this loss of growth advantage in the optimized hygiene group, since tail biting negatively impacts ADG (van Staaveren et al., 2021). However, hygiene-associated gut microbiota disruption at birth which persists thereafter, as shown by Law et al. (2021), was most likely the main causal factor. While Law et al. showed improved growth in pigs born into a disinfected farrowing environment, as we did, they only followed pigs for 6 weeks PW (beyond which our growth advantage was lost). Thereafter, it could be hypothesized that these pigs with reduced immunocompetence due to reduced microbial exposure early in life would be more susceptible to infection when introduced to a less sanitary environment as would have been the case in the finisher accommodation used in the current study. As no other studies to date have investigated the residual effects of improved farrowing room hygiene, no direct comparisons can be made with the results from the current study. However, it is likely that in order to see residual effects on growth to slaughter weight, weaner and finisher accommodation hygiene should also be improved.

## CONCLUSIONS

Implementation of an optimized hygiene routine in farrowing accommodation reduced the bacterial load in the farrowing pens, which led to a reduction in diarrhea prevalence and clinical cases of disease during the suckling period, with antibiotic and anti-inflammatory usage reduced as a result. This is important, given the current legislative drivers to reduce medication usage and to improve health and welfare in pigs. Furthermore, weaning weight increased in pigs born into more hygienic pens, and residual growth effects were seen PW. However, this growth benefit was not retained through to slaughter. This is thought to be due to reduced immunocompetence in pigs born into the optimized hygiene environment, resulting in reduced growth when pigs were housed in less sanitary conditions during this period. Implementation of the optimized hygiene routine has a cost implication and requires additional labor, as well as sufficient farrowing accommodation to allow pens to dry. However, a maximum of 3 d of drying appears adequate and avoids potential re-contamination of pens. Overall, to our knowledge, this is the first study to show reduced medication usage as a result of implementing an improved farrowing accommodation hygiene routine and one of the first to show improved growth. Based on these results, an easily implementable sanitization protocol with demonstrable benefits can now be provided to farmers, although a detailed cost-benefit analysis is required in order to encourage a change in farmer behavior.

## Supplementary Material

txae095_suppl_Supplementary_Materials
