# Supplementary material for: Effect of implementing an effective farrowing accommodation hygiene routine on clinical cases of disease, medication usage, and growth in suckling and weaned pigs
Source: Transl Anim Sci. 2024 Jun 14;8:txae095. doi: 10.1093/tas/txae095 (PMC11263928; doi:10.1093/tas/txae095)
Supplement: txae095_suppl_Supplementary_Materials [file txae095_suppl_supplementary_materials.docx]

**SUPPLEMENTARY MATERIALS**

Running head: Farrowing accommodation hygiene

**Effect of implementing an effective farrowing accommodation hygiene routine on clinical cases of disease, medication usage and growth in suckling and weaned pigs.**

**K.M. Halpin*†, P.G. Lawlor*^2^, E.A. Arnaud*^,^†, J. Teixé-Roig*^,§^, J.V. O’ Doherty‡, T. Sweeney**¶**, T.M. O’ Brien#, G.E. Gardiner†**

*Teagasc, Pig Development Department, Animal and Grassland Research and Innovation Centre, Moorepark, Fermoy, County Cork, Ireland.

† Eco-Innovation Research Centre, Department of Science, South East Technological University, Waterford, Ireland.

**‡** School of Agriculture and Food Science, University College Dublin, Belfield, Dublin 4, Ireland.

¶School of Veterinary Medicine, University College Dublin, Belfield, Dublin 4, Ireland.

^§^Food Technology Department, University of Lleida, Lleida, Spain.

 #Teagasc, Food Research Centre, Moorepark, Fermoy, County Cork, Ireland.

^1^ The PigNutriStrat project is funded by the Irish Department of Agriculture, Food and the Marine’s Competitive Research Funding Programmes (Grant no: 2019R518). The authors would like to thank Tomas Ryan, Aisling Holmes, David Clarke, John Condon, John Heffernan, Pat Magnier, John Walsh, Shane Kenny, Kieran Keane and Dan O’ Donovan of the Pig Development Department at Teagasc, Moorepark and placement student Jane Kelleghan of Munster Technological University for assistance in conducting the animal study. We also thank Jimmy Flynn of Teagasc, Moorepark for advice and assistance with microbiological analysis.

^2^ Corresponding author: [peadar.lawlor@teagasc.ie](mailto:peadar.lawlor@teagasc.ie)

**Table S1 Effect of hygiene routine on sow weights, back fat thickness, injections and medication usage^1^.**

|  | Basic^2^ | Optimised^3^ | SEM | P-value^4^ |
| --- | --- | --- | --- | --- |
| Number of sows | 22 | 25 |  |  |
| Sow body weight, kg |  |  |  |  |
| Day 110 of gestation | 279.9 | 287.5 | 2.50 | 0.89 |
| Weaning | 243.4 | 239.5 | 2.50 | 0.21 |
| Service | 235.0 | 233.1 | 2.69 | 0.60 |
| Sow back fat, mm |  |  |  |  |
| Day 110 of gestation | 14.7 | 14.7 | 0.52 | 0.41 |
| Weaning | 12.1 | 11.8 | 0.56 | 0.96 |
| Service | 13.0 | 12.4 | 0.55 | 0.64 |
| Injections and medication |  |  |  |  |
| Number of injections per litter | 0.7 | 0.8 | 0.32 | 0.88 |
| Antibiotic usage per sow, ml | 12.5 | 14.4 | 5.75 | 0.82 |
| Anti-inflammatory usage per sow, ml | 2.9 | 3.2 | 1.31 | 0.88 |

^1^Least square mean values with their pooled standard errors of the mean (SEM)

^2^Basic hygiene routine = washing of pens with cold water, no use of detergent or disinfectant and minimal drying time (40 h).

^3^Optimised hygiene routine = pre-soak with cold water for ≤18 h, detergent application (contact time of 20 min), washing of pens with cold water, application of a disinfectant 24 h later and pens allowed to dry for 6 days.

^4^Mean values were significantly different between treatments when *P* ≤ 0.05.

**Table S2 Effect of hygiene routine on post-weaning diarrhoea incidence, clinical cases of disease, injections and medication usage^1^.**

|  | Basic^2^ | Optimised^3^ | SEM | P-value^4^ |
| --- | --- | --- | --- | --- |
| Number of pens | 9 | 12 |  |  |
| Diarrhoea prevalence, % (weaning to day 28 post-weaning)^5^ day)weaningweweaningweaning)^1^ | 7.3 | 2.4 | - | - |
| Clinical cases of disease, number/pen^6^ | 1.5 | 1.3 | 0.45 | 0.70 |
| Number of injections per pen | 9.4 | 6.3 | 2.75 | 0.43 |
| Antibiotic usage per pen, ml | 10.8 | 8.0 | 3.88 | 0.60 |
| Anti-inflammatory usage per pen, ml | 3.8 | 2.9 | 1.25 | 0.60 |

^1^Least square mean values with their pooled standard errors of the mean (SEM)

^2^Basic hygiene routine = washing of pens with cold water, no use of detergent or disinfectant and minimal drying time (40 h).

^3^Optimised hygiene routine = pre-soak with cold water for ≤18 h, detergent application (contact time of 20 min), washing of pens with cold water, application of a disinfectant 24 h later and pens allowed to dry for 6 days.

^4^Mean values were significantly different between treatments when *P* ≤ 0.05.

^5^A faecal score of 2 or greater for each pen was considered indicative of diarrhoea at each time point from weaning to day 28 post-weaning. The overall prevalence was reported for the post-weaning period.

^6^Number of pigs per pen requiring treatment with medication during the post-weaning period on one or more occasions.

**
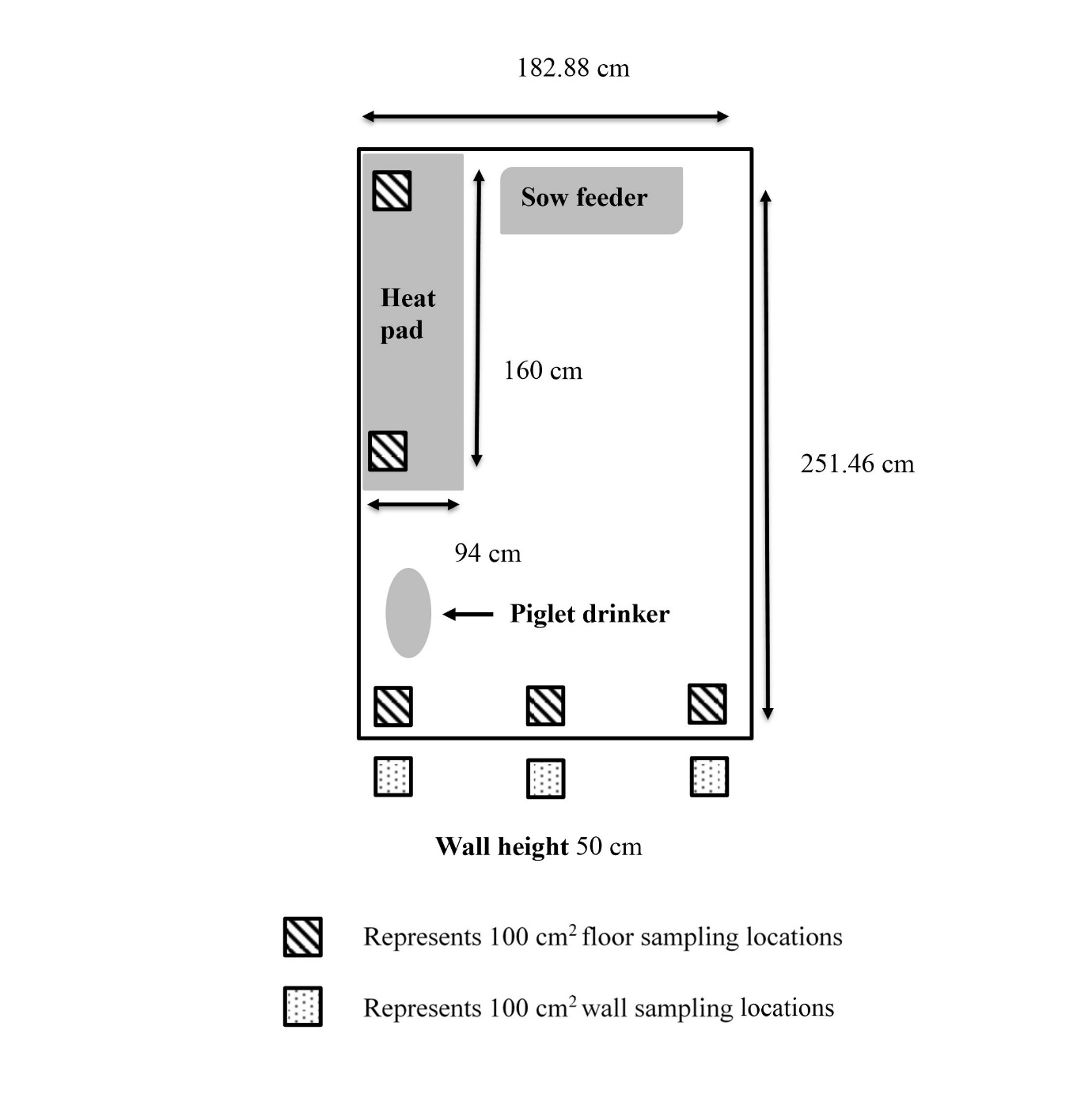
**

**Figure S1** Farrowing pen dimensions and sampling locations
